# Comparative Adverse Kidney Outcomes in Women Receiving Raloxifene and Denosumab in a Real-World Setting

**DOI:** 10.3390/biomedicines10071494

**Published:** 2022-06-24

**Authors:** Hsin-Wei Chen, Chien-Ning Hsu, Yueh-Ting Lee, Chung-Ming Fu, Shih-Wei Wang, Chiang-Chi Huang, Lung-Chih Li

**Affiliations:** 1 Division of Nephrology, Department of Internal Medicine, Kaohsiung Chang Gung Memorial Hospital and Chang Gung University College of Medicine, Kaohsiung 833, Taiwan; sunset1275@hotmail.com (H.-W.C.); yuai@cgmh.org.tw (Y.-T.L.); bugfu0624@gmail.com (C.-M.F.); herme381981@gmail.com (C.-C.H.); 2 Department of Pharmacy, Kaohsiung Chang Gung Memorial Hospital and Chang Gung University College of Medicine, Kaohsiung 833, Taiwan; chien_ning_hsu@hotmail.com (C.-N.H.); ntucom@cgmh.org.tw (S.-W.W.); 3 School of Pharmacy, Kaohsiung Medical University, Kaohsiung 807, Taiwan; 4 Institute for Translational Research in Biomedicine, Kaohsiung Chang Gung Memorial Hospital and Chang Gung University College of Medicine, Kaohsiung 833, Taiwan

**Keywords:** acute kidney injury, chronic kidney disease, denosumab, raloxifene, osteoporosis

## Abstract

Both osteoporosis and kidney diseases are common and intercorrelate to increase morbidity and mortality in elderly women. This study aimed to compare adverse kidney outcome between women initiated with denosumab and a matched group of raloxifene initiators using propensity score matching methods in a large healthcare delivery system in Taiwan. The risks of adverse kidney outcomes were estimated using Cox proportional hazard regression and the change in kidney function over time was analyzed using the linear mixed model. A total of 9444 (4722 in each group) women were identified who matched the inclusion criteria between January 2003 and December, 2018. Denosumab use was significantly associated with higher risk of eGFR decline ≥ 30% from baseline than raloxifene use (aHR: 1.26; 95% CI: 1.16–1.36, *p* < 0.0001). The mean change in eGFR over time was 1.24 mL/min/1.73 m^2^ per year in the denosumab group and 0.45 mL/min/1.73 m^2^ per year in the raloxifene group (*p* = 0.0004). However, the risks of acute kidney injury (10.53%) and chronic dialysis (0.66%) in this study cohort were not significantly different for the two anti-osteoporosis treatments. Close monitoring of the residual kidney function and treatment effect is needed in those with denosumab.

## 1. Introduction

As the world is experiencing an unprecedented rise in the elderly population, the prevalence of many age-related disorders, including osteoporosis and chronic kidney disease (CKD), is also increasing. Previous studies have demonstrated that patients with kidney dysfunction show reduced bone mineral density (BMD) and an increased risk of fracture [1,2,3]. In patients with CKD, the regulatory system of calcium and phosphate is altered, resulting in disorders of bone remodeling known as CKD-mineral and bone disorders (CKD-MBD). The co-existence of CKD and osteoporosis has become challenging to manage and a huge burden on healthcare systems worldwide [4].

Different anti-osteoporotic agents targeting various stages of bone remodeling have been developed in recent decades, including bisphosphonates, selective estrogen receptor modulators (SERM), and inhibitors of the receptor activator of nuclear factor-κB ligand (RANKL). Many of these agents are metabolized by the kidneys, and it has been suggested that they require dose adjustment in patients with renal impairment [5]. Although bisphosphonates are most frequently used, they are not recommended in patients with an estimated glomerular filtration rate (eGFR) of <30 mL/min/1.73 m^2^ [6]. New anti-osteoporotic agents, including raloxifene and denosumab, are frequently used in women with osteoporosis and advanced CKD [7,8].

Raloxifene, a second-generation SERM, is a common treatment option for women with osteoporosis. By regulating transforming factor-β3 gene expression and suppressing interleukin-6 promoter activity, raloxifene maintains bone mass by inhibiting the differentiation and bone-resorptive activities of osteoclasts [7,9]. The Multiple Outcomes of Raloxifene Evaluation (MORE) study demonstrated raloxifene’s ability to increase bone mineral density in the spine and femoral neck and reduce the risk of vertebral fracture in postmenopausal women with osteoporosis [10]. In the post hoc analysis of the MORE study [11], a possible renoprotective effect was demonstrated by comparing postmenopausal women taking placebo and raloxifene (60 mg or 120 mg) over the course of 3 years, which showed a slower decrease in mean eGFR in the raloxifene groups (mean decline of 0.34 mL/min/1.73 m^2^ per year; *p* < 0.0001). However, the interpretation of this result in the general population may be limited due to the exclusion of patients with a serum creatinine level above 2.5 mg/dL in this study.

Denosumab is a human monoclonal antibody that acts as an osteoprotegerin (OPG) mimic against the receptor activator, RANKL. By interrupting the binding of RANKL to RANK, denosumab suppresses osteoclastogenesis, decreases bone reabsorption, and increases bone density [12,13]. In the Fracture Reduction Evaluation of Denosumab in Osteoporosis Every 6 Months (FREEDOM) trial, the use of denosumab significantly reduced the risk of vertebral, nonvertebral, and hip fractures in women with osteoporosis [13]. Denosumab is metabolized by the reticuloendothelial system as an antibody, with minimal renal filtration and excretion [14,15]. Hence, denosumab is generally considered to have neutral effects on renal function in patients with CKD [16].

Along with bisphosphonates, both denosumab and raloxifene are recommended treatment agents in guidelines and are reimbursed by the Taiwan National Health Insurance program [17]; however, their long-term effects on renal function have not been studied in as much depth. In our previous study comparing the effects of denosumab and alendronate on cardiovascular and renal outcomes, an increased risk of deterioration in renal function in patients with renal insufficiency was observed in the denosumab group after 5-year follow-up [18]. Hence, this study aimed to further evaluate the various kidney outcomes of denosumab and raloxifene users in women with osteoporosis.

## 2. Materials and Methods

### 2.1. Data Source

This retrospective cohort study used electronic health record (EHR) data from the Chang Gung Research Database (CGRD) of the healthcare delivery system of Chang Gung Memorial Hospital (CGMH) in Taiwan. Briefly, the CGRD contains individual patient-level EHR from the CGMH, providing 10–12% of health services reimbursed by Taiwan National Health Insurance (NHI) program in 2018 [19]. The data generalizability of CGRD has been validated in some disease populations [20,21], and the laboratory results have been used in kidney [18,22,23] and heart studies [24]. The CGRD data from January 2003 to December 2018 were used for the analysis. This study was approved by the Institutional Review Board of Chang Gung Medical Foundation in Taipei, Taiwan (Permit number: 201900898B0).

### 2.2. Study Cohort

**Figure 1:**
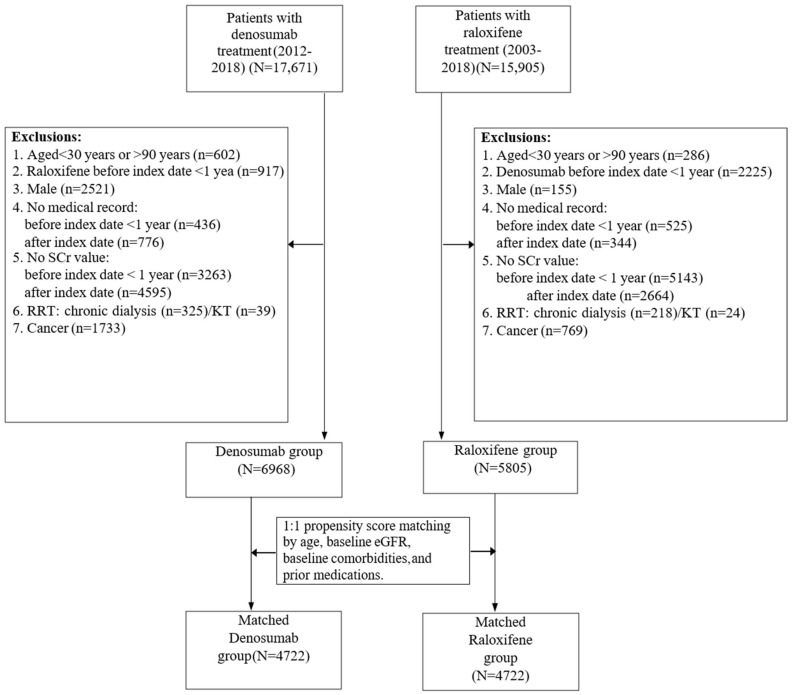
Flowchart and patient selection process. Index date: the date the patient was initiated with denosumab or raloxifene; ACEI: Angiotensin converting enzyme inhibitor; ARB: Angiotensin receptor blockers; SCr: serum creatinine; KRT: kidney replacement therapy; KT: kidney transplant; eGFR: estimated glomerular filtration rate.

Using the CGRD, we first identified new users of denosumab and raloxifene in the outpatient department. To ensure that new users of denosumab and raloxifene were included, patients without medical records for at least one year before the start of treatment and those who had ever been treated with counterpart therapy were excluded. Other inclusion criteria were patients aged between 30 and 90 years at the index date (the start of denosumab or raloxifene therapy), with at least one serum creatinine (SCr) baseline measurement. For kidney outcome assessment, patients were excluded due to a lack of medical encounters after the index date, and if they had undergone kidney replacement therapy (KRT) or cancer diagnosis before the index date. Because most patients in the raloxifene group were female, male patients were excluded from the analysis (Figure 1). Further codes and operational definitions are detailed in the Supplementary Materials, Table S1.

Both denosumab and raloxifene are reimbursed by the Taiwan NHI program as monotherapy for confirmed osteoporosis, which is defined as a T score of bone mineral density ≤−2.5 standard deviation (SD) measured using dual-energy X-ray absorptiometry (DXA) scanning with a history of one or more spinal or femoral fracture, or osteopenia (−2.5 SD < T score of bone mineral density < −1.0 SD by DXA scan) with two or more spinal or femoral fractures. Denosumab (60 mg/mL/syringe) is recommended to be administered at one unit per 6 months, whereas administration of raloxifene (60 mg/tab) is recommended at one unit per day. To quantify the intensity of treatment exposure, the proportion of days covered (PDC) was defined as ([number of syringes prescribed/expected total number of syringes in the follow-up] × 100) for denosumab users. The PDC for raloxifene users was defined as ([sum of days covered by raloxifene/number of follow-up days] × 100) [25].

### 2.3. Outcomes and Follow-Up

The outcomes of interest were acute kidney injury (AKI), time to ≥30% estimated glomerular filtration rate (eGFR) reduction from baseline measurement [26], and initiation of chronic dialysis or KRT. SCr-based AKI was defined by the Kidney Disease Improving Global Outcomes (KDIGO) stage as an increase in SCr of ≥0.3 mg/dL within 48 h. Stage 1 was classified as an increase in SCr of 1.5 to 1.9 times the baseline value, which is known or presumed to have occurred within the prior 7 days; an increase in SCr of 2.0 to 2.9 times the baseline value was classified as stage 2; and an increase in SCr to 3.0 times the baseline value, an increase in SCr to ≥4.0 mg/dL, or the initiation of renal replacement therapy were classified as stage 3 [27].

eGFR was calculated using the Taiwan version of the abbreviated Modification of Diet in Renal Disease equation (eGFR = 175 × SCr^−1.154^ × age^−0.203^ × 0.742 (if female)) [28]. The follow-up time was divided into 3-month intervals, and time-interval averages of eGFR in each time interval were used to assess the change in eGFR over time (baseline eGFR, the last averaged eGFR) between the treatment groups. Using the as-treated analytic approach, patients were followed from the index date to the occurrence of study outcomes, treatment discontinuation, a switch to alternative treatment, in-hospital death, last encounter date, or the last date of the study period in the CGRD (31 December 2018), whichever came first.

### 2.4. Study Covariates

The following study covariates were measured at baseline as potential confounders to be adjusted in the analyses: age, gender, eGFR, other medications with ≥28 days use (e.g., non-steroid anti-inflammatory drugs (NSAID), aspirin, antithrombotic medications, osteoporosis medications), and the Charlson Comorbid Index [29], which were measured in the year before the index date (Supplementary Materials, Table S1).

It is known that AKI can accelerate kidney function deterioration [30] and potentially increase the risk of eGFR reduction. AKI episodes that occurred before the first event of eGFR reduction of ≥30% were considered as time-varying covariates in the follow-up.

### 2.5. Statistical Analysis

Propensity score (PS) matching was applied to balance the differences in baseline demographic and clinical characteristics between the denosumab and raloxifene groups. The individual PS of patients receiving denosumab or raloxifene was estimated using logistic regression. New users of denosumab and raloxifene were matched at a 1:1 ratio using the greedy algorithm of the PS matching method [31]. The comparison of baseline characteristics between the denosumab and raloxifene groups was performed using the standardized mean difference (SDM), and an SDM of <0.1 was considered as no meaningful difference [32].

Among the matched denosumab or raloxifene new users, we used the Cox proportional model to estimate the adjusted hazard ratios (aHR) of the study outcomes between denosumab and raloxifene users, controlling for time-varying covariates (e.g., an AKI episode that occurred earlier than other kidney outcomes). A linear mixed-effect model was used to estimate the difference in average changes in eGFR over time between denosumab and raloxifene users. To assess the heterogeneous effects of denosumab (versus raloxifene) by different baseline characteristics, stratified analyses were performed in the matched cohorts by age < 65 years (vs. age ≥65 years) and baseline eGFR groups (≥60, 30–59.9, and <30 mL/min/1.73 m^2^). A two-tailed test (*p* value < 0.05) was considered statistically significant. All statistical analyses were performed using SAS 9.4 (SAS Institute, Cary, NC, USA).

## 3. Results

### 3.1. Patient Characteristics

We identified 12,773 patients (6968 in the denosumab group and 5805 in the raloxifene group) who met the inclusion criteria. The selection process for the study cohort is shown in Figure 1. Before PS matching, denosumab new users were significantly older than raloxifene users (73.92 ± 9.56 versus 70.31 ± 10.69 years old, *p* < 0.001). The proportion of patients with a baseline eGFR of <30 mL/min/1.73 m^2^ was lower in denosumab users than in raloxifene users (7.89 versus 8.56%).

Compared with patients in the raloxifene group, denosumab users had more chronic diseases, such as kidney disease, hypertension, and hyperlipidemia. NSAID prescription use was higher in denosumab users than that in raloxifene users (72.85 versus 68.6%); but calcium (24.31 versus 35.92%), vitamin D (16.79 versus 25.63%), and calcitonin (0.72 versus 3.15%) use was lower in denosumab users compared with raloxifene users. The differences in the baseline demographic and clinical characteristics are presented in Table 1. In the PS-matched cohort (*n* = 4722 in each group), the average age was 72 years. The baseline demographic and clinical characteristics were well balanced between the denosumab and raloxifene users (SMD < 0.1) (Table 1).

### 3.2. Adverse Kidney Outcomes

During the 6-year follow-up period, the cumulative incidences of AKI were 10.53% (*n* = 497) and 13.68% (*n* = 646) in the denosumab and raloxifene groups, respectively. The incidence rate of eGFR decline to ≥ 30% of the baseline value was 30.45% (*n* = 1438) in the denosumab group and 32.78% (*n* = 1548) in the raloxifene group (Table 2). Overall, 0.66% (*n* = 61) of the patients required chronic dialysis therapy.

#### 3.2.1. Acute Kidney Injury

**Figure 2:**
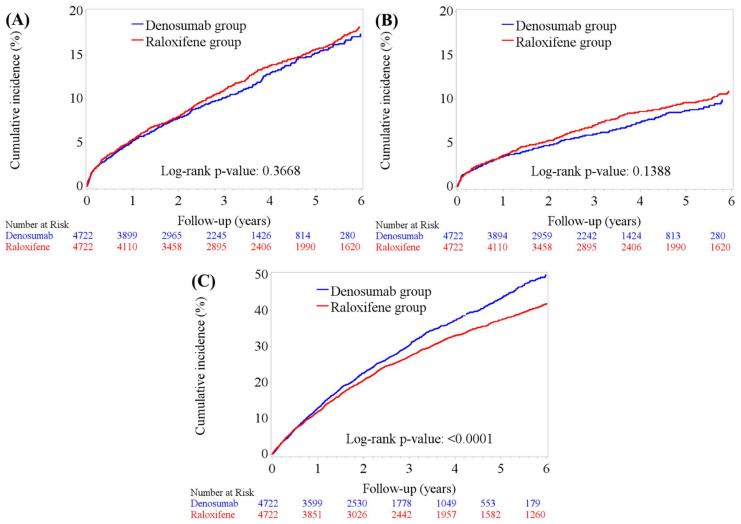
Cumulative incidence of kidney outcomes in the denosumab and raloxifene groups. (**A**) Any AKI event (log-rank test, *p* = 0.3668); (**B**) advanced AKI (KDIGO stage 2, 3 and dialysis) (log-rank test, *p* = 0.1388); (**C**) eGFR decline ≥30% from baseline (log-rank test, *p* < 0.001) (*n* = 9444).

**Figure 3:**
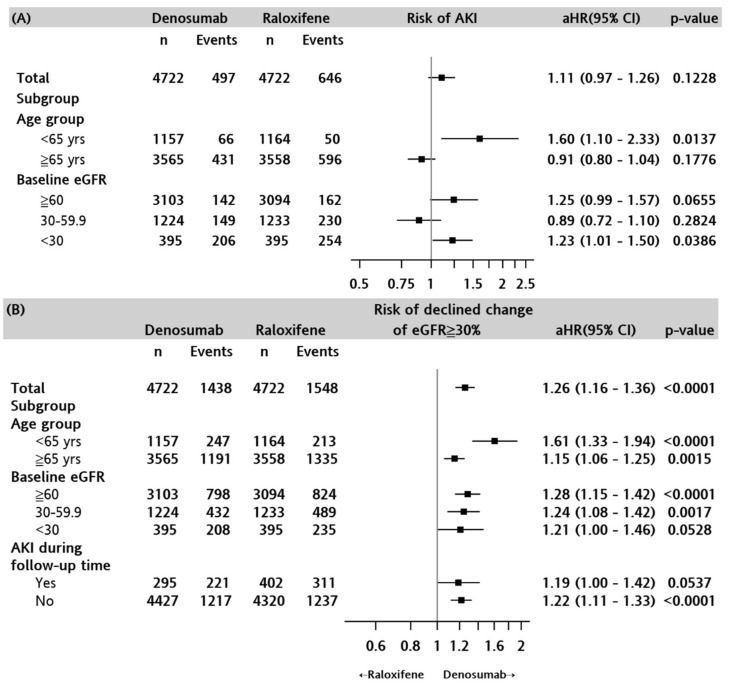
Cox regression model of (**A**) AKI and (**B**) eGFR decline ≥ 30% from baseline between denosumab and raloxifene groups. AKI: Acute kidney injury; aHR: adjusted hazard ratio.

As shown in Figure 2A, the cumulative incidence of AKI was similar in both the treatment groups (log-rank test, *p* = 0.3668), as was the incidence of advanced AKI (Stage 2/3 or acquired dialysis) (log-rank test, *p* = 0.1388). The results of Cox regression analyses showed that denosumab was not significantly associated with any AKI risk compared with raloxifene (aHR, 1.11; 95% CI, 0.97-1.26; *p* = 0.1228), and the association was weaker for advanced AKI (aHR, 1.05; 95% CI, 0.88-1.24; *p* = 0.6028) after controlling for baseline demographic, clinical characteristics, and prior medication use (Supplementary Materials, Table S2). In the stratified analyses (Figure 3A), denosumab use was associated with a higher risk of AKI than raloxifene in patients aged < 65 years (aHR, 1.60; 95% CI, 1.10–2.33; *p* = 0.0137), as well as in patients with a baseline eGFR of <30 mL/min/1.73 m^2^ (aHR, 1.23; 95% CI, 1.01–1.50; *p* = 0.0386).

#### 3.2.2. eGFR Reduction ≥ 30%

As shown in Figure 2C, the cumulative incidence of eGFR decline ≥ 30% was significantly higher in the denosumab group than in the raloxifene group over the study period (log-rank test, *p* < 0.001). Cox regression analysis showed that denosumab use was significantly associated with a higher risk of eGFR reduction than raloxifene was (aHR, 1.26; 95% CI: 1.16–1.36, *p* < 0.0001) (Figure 3B). The increased hazard of eGFR reduction ≥ 30% was higher in the denosumab group in patients of all ages but was more profound in those aged < 65 years (aHR, 1.61; 95% CI, 1.33–1.94, *p* < 0.0001). Reduction of eGFR >30% was also greater in the denosumab group (versus raloxifene) compared with baseline eGFR >60 (aHR, 1.28; 95% CI, 1.15–1.42; *p* <0.001) and 30–59.9 mL/min/1.73 m^2^ (aHR, 1.24; 95% CI, 1.08–1.42; *p* = 0.0017), but was less significant in patients with a baseline eGFR of <30 mL/min/1.73 m^2^ (aHR, 1.21; 95% CI, 1.00–1.46; *p* = 0.0528). The risk of an eGFR reduction of ≥30% did not differ between treatment groups in patients who had an episode of any AKI (aHR, 1.19; 95% CI, 1.00–1.42; *p* = 0.0537).

### 3.3. Changes in eGFR over Time

The median yearly decline in eGFR from baseline in the denosumab group was 1.24 (25th, 75th percentile, −3.05, 5.67) ml/min/1.73 m^2^ and 0.45 (−3.02, 4.68) ml/min/1.73 m^2^ in the raloxifene group. Among patients with an eGFR >60 mL/min/1.73 m^2^ at baseline, the median yearly decline in eGFR in the denosumab group was 2.04 (−2.41, 7.17) ml/min/1.73 m^2^, which was greater than that of raloxifene users 0.75 (−2.91, 5.76) ml/min/1.73 m^2^. Figure S1 shows the eGFR trajectories of denosumab and raloxifene users over 72 months. Compared with raloxifene users, denosumab use was associated with a statistically significant decline in eGFR over time (adjusted β = −0.33; 95% CI, −0.36 to −0.29 mL/min/1.73 m^2^, *p <* 0.0001) after adjusting for age, baseline eGFR, PDC and AKI event (Table 3).

## 4. Discussion

In the current study, we observed that denosumab had an additional risk of eGFR reduction of ≥30% from baseline compared with raloxifene. Patients aged < 65 years who were treated with denosumab had an increased risk of eGFR reduction ≥ 30% and AKI development. The magnitude of the relative risk of adverse kidney events between denosumab and raloxifene was somewhat different according to baseline eGFR. To the best of our knowledge, this is the first study to comprehensively evaluate the kidney safety of denosumab compared with raloxifene in a female population.

AKI is characterized by an abrupt decline in glomerular filtration rate, which is associated with high mortality and morbidity [33]. AKI is also a distinct risk factor for CKD progression [34]. Although the effects of denosumab and raloxifene on osteoporosis and the prevention of osteoporosis-related bone fractures have been effectively demonstrated [10,13,35], their effects on AKI remain unclear. The increase in AKI events observed in patients with an eGFR of <30 mL/min/1.73 m^2^ in the denosumab group is consistent with current knowledge, as the underlying renal function worsens and patients are more vulnerable to acute kidney injury [30]. However, the result of a higher AKI rate in younger patients (age < 65 years) among denosumab users is somewhat elusive. The prescription of medication dramatically increases in the elderly compared to that in younger patients [36]. Despite the fact that patients older than 65 years had higher rates of total AKI than younger patients (Supplementary Materials, Table S2), the possibility of AKI resulting from polypharmacy may escalate in elderly people [37]. In younger patients, the results may be unmasked and signify the true difference between the effects of denosumab and raloxifene, with fewer confounding factors. Further studies are warranted to verify these findings.

A decrease in eGFR of ≥30% from baseline has been proposed as an indicator of CKD progression [38]. As a higher rate of eGFR decline was observed in the denosumab group, this may translate to the possible worsening of CKD progression in denosumab users. This finding was consistent with a previous study comparing denosumab with alendronate, and the increase in renal function decline in the denosumab group was also observed in patients with poor baseline renal function with an eGFR of <60 mL/min/1.73 m^2^ (aHR, 1.5; 95% CI 1.09–2.07; *p* = 0.0132) [18]. However, these results were in contrast to those of another FREEDOM extension study, which revealed no significant change in CKD stage between denosumab and placebo use [16]. The majority (>60%) of patients in the FREEDOM extension study had relatively preserved renal function, namely, in CKD stages 2 and 3a. In addition, the renal function change in the FREEDOM extension study was roughly classified by CKD stage difference rather than by direct change in eGFR, all of which may result in an underestimated decline in renal function. It is also possible that different populations or ethnic differences could result in the different results from our study.

As an SERM, raloxifene may exert renoprotective effects similar to estrogen, as seen in our subgroup analysis, and almost all patients using raloxifene showed a trend of decreasing CKD progression, despite differences in age, baseline eGFR, and AKI occurrence. The trend of mean eGFR also remained steady in the raloxifene group, whereas eGFR in the denosumab group declined progressively during the 6-year follow-up. This may confirm the prior hypothesis of the MORE post hoc study [11]. However, the mechanism underlying its possible protective effect warrants further study.

Multiple mechanisms have been proposed in prior studies investigating the role of estrogen and estrogen receptors (ERα and ERβ) in kidney diseases [39], including antioxidative stress of silent mating-type information regulator 2 homolog 1 (SIRT1) [40], possible antifibrotic effects, and protection from the damaging effects of testosterone [41] in different animal models of AKI and CKD. Previous studies have demonstrated that premenopausal women who underwent bilateral oophorectomy were at a higher risk of developing CKD, suggesting a renoprotective effect of estrogen [42]. As an SERM, raloxifene may exert its effects via estrogen agonists in renal tissues. It was observed in an animal study that raloxifene decreased cisplatin-induced nephrotoxicity when administered before cisplatin treatment owing to its anti-inflammatory effects [43]. Moreover, raloxifene attenuates albuminuria in streptozotocin-induced diabetic rats [44].

Compared with denosumab, raloxifene seems to be a better choice for long-term kidney function. Further studies on the long-term safety of denosumab and raloxifene should include measures of adverse kidney outcomes as the management of osteoporosis related to CKD-MBD warrants further evidence-based studies. The confirmation of different outcomes based on different CKD stages will also benefit the selection of future treatment choices.

Although this large cohort of female patients taking osteoporosis medications enables us to detect and compare the relative risk of adverse kidney outcomes of denosumab and raloxifene, this study has some limitations. First, we used the Taiwan NHI program reimbursement criteria to identify denosumab and raloxifene users. Bone mineral density, T-score, and baseline mineral and bone disorders were not assessed, which may lead to an underestimation of the baseline risk of adverse kidney outcomes in both treatment groups, and may not represent the general patient population. Second, unmeasured confounders such as volume status and major surgeries associated with AKI development were not assessed for AKI outcomes. Third, the prescription pattern or frequency of eGFR monitoring could affect the rate of adverse kidney outcomes. Caution should be exercised when comparing kidney safety across different health care systems.

## 5. Conclusions

In conclusion, the baseline eGFR and patient age were associated with the relative risk of AKI and long-term kidney outcomes between denosumab and raloxifene. Baseline kidney function assessment and close monitoring are essential for planning treatment initiation and shared decision-making regarding treatment choice in women with osteoporosis who are at a high risk of kidney function progression. Raloxifene might be a better alternative to denosumab when long-term kidney outcomes are a priority.

## Figures and Tables

**Table 1:** Characteristics of study cohort. PSM: propensity score matching; SMD: standardized mean difference, SMD < 0.1 was considered as no sign of imbalance; NSAID = Nonsteroidal anti-inflammatory drugs; ACEI: Angiotensin converting enzyme inhibitor; ARB: Angiotensin receptor blockers.

	Without PSM				PSM			
	Overall (*n*)	Denosumab (*n* = 6968)	Raloxifene (*n* = 5805)	SMD	Overall (*n*)	Denosumab (*n* = 4722)	Raloxifene (*n* = 4722)	SMD
		*n* (%)	*n* (%)			*n* (%)	*n* (%)	
Baseline eGFR, mg/min/1.73 m^2^								
≧90	3331	1961 (28.14)	1370 (23.60)	0.104	2404	1202 (25.46)	1202 (25.46)	0.000
60–89.9	5082	2616 (37.54)	2466 (42.48)	0.101	3793	1901 (40.26)	1892 (40.07)	0.004
45–59.9	1925	1037 (14.88)	888 (15.30)	0.012	1459	723 (15.31)	736 (15.59)	0.008
30–44.9	1394	804 (11.54)	590 (10.16)	0.044	998	501 (10.61)	497 (10.53)	0.003
15–29.9	727	394 (5.65)	333 (5.74)	0.004	539	270 (5.72)	269 (5.70)	0.001
<15	314	156 (2.24)	158 (2.72)	0.031	251	125 (2.65)	126 (2.67)	0.001
Charlson comorbidity index condition								
Acute myocardial infarction	137	82 (1.18)	55 (0.95)	0.022	98	51 (1.08)	47 (1.00)	0.008
Congestive heart failure	737	438 (6.29)	299 (5.15)	0.049	514	258 (5.46)	256 (5.42)	0.002
Peripheral vascular diseases	235	137 (1.97)	98 (1.69)	0.021	164	76 (1.61)	88 (1.86)	0.020
Cerebral vascular accident	1704	1043 (14.97)	661 (11.39)	0.106	1179	600 (12.71)	579 (12.26)	0.014
Dementia	624	404 (5.80)	220 (3.79)	0.094	406	204 (4.32)	202 (4.28)	0.002
Pulmonary disease	1499	878 (12.60)	621 (10.70)	0.059	1090	551 (11.67)	539 (11.41)	0.008
Connective tissue disorder	604	357 (5.12)	247 (4.25)	0.041	443	220 (4.66)	223 (4.72)	0.003
Peptic ulcer	2890	1584 (22.73)	1306 (22.50)	0.006	2124	1065 (22.55)	1059 (22.43)	0.003
Liver diseases	1530	889 (12.76)	641 (11.04)	0.053	1125	579 (12.26)	546 (11.56)	0.022
Diabetes	3384	1974 (28.33)	1410 (24.29)	0.092	2468	1238 (26.22)	1230 (26.05)	0.004
Diabetes complications	1098	664 (9.53)	434 (7.48)	0.074	775	385 (8.15)	390 (8.26)	0.004
Paraplegia	144	82 (1.18)	62 (1.07)	0.010	112	62 (1.31)	50 (1.06)	0.024
Renal disease	1438	929 (13.33)	509 (8.77)	0.146	911	453 (9.59)	458 (9.70)	0.004
Severe liver diseases	55	33 (0.47)	22 (0.38)	0.015	44	24 (0.51)	20 (0.42)	0.012
Metastatic cancer	4	3 (0.04)	1 (0.02)	0.015	2	1 (0.02)	1 (0.02)	0.000
Hypertension	6659	3942 (56.57)	2717 (46.80)	0.196	4740	2374 (50.28)	2366 (50.11)	0.003
Hyperlipidemia	4014	2458 (35.28)	1556 (26.80)	0.184	2745	1373 (29.08)	1372 (29.06)	0.001
Thyroid function abnormal	353	226 (3.24)	127 (2.19)	0.065	223	109 (2.31)	114 (2.41)	0.007
Obstructive sleep apnea	342	261 (3.75)	81 (1.40)	0.149	167	87 (1.84)	80 (1.69)	0.011
Prior medication								
Oral anticoagulants	373	256 (3.67)	117 (2.02)	0.100	220	110 (2.33)	110 (2.33)	0.000
Anti-platelet	2787	1655 (23.75)	1132 (19.50)	0.103	1974	1003 (21.24)	971 (20.56)	0.017
Aspirin	2080	1225 (17.58)	855 (14.73)	0.078	1487	755 (15.99)	732 (15.50)	0.013
Statins	3129	1997 (28.66)	1132 (19.50)	0.215	2130	1071 (22.68)	1059 (22.43)	0.006
Fibrates	310	200 (2.87)	110 (1.89)	0.064	200	105 (2.22)	95 (2.01)	0.015
Other lipid-lowering agents	85	66 (0.95)	19 (0.33)	0.078	43	24 (0.51)	19 (0.40)	0.016
Anti-diabetics	2792	1632 (23.42)	1160 (19.98)	0.084	2034	1028 (21.77)	1006 (21.30)	0.011
ACEI/ARB/Aliskiren	4254	2589 (37.16)	1665 (28.68)	0.181	2966	1503 (31.83)	1463 (30.98)	0.018
Diuretics	819	396 (5.68)	423 (7.29)	0.065	582	295 (6.25)	287 (6.08)	0.007
Alendronate	1714	946 (13.58)	768 (13.23)	0.010	1290	654 (13.85)	636 (13.47)	0.011
Teriparatide	341	194 (2.78)	147 (2.53)	0.016	256	127 (2.69)	129 (2.73)	0.003
Calcitonin preparations	233	50 (0.72)	183 (3.15)	0.177	103	50 (1.06)	53 (1.12)	0.006
Calcium	3779	1694 (24.31)	2085 (35.92)	0.255	2833	1414 (29.94)	1419 (30.05)	0.002
Vitamin D	2658	1170 (16.79)	1488 (25.63)	0.218	2014	1003 (21.24)	1011 (21.41)	0.004
NSAID	9058	5076 (72.85)	3982 (68.60)	0.094	6655	3331 (70.54)	3324 (70.39)	0.003
		Mean (SD)				Mean (SD)		
Age at the index date, years	12,773	73.92 (9.56)	70.31 (10.69)	0.356	9444	71.94 (9.86)	71.95 (9.86)	0.001
Baseline eGFR, ml/min/1.73 m^2^	12,773	73.10 (31.26)	71.10 (29.75)		9444	72.49 (30.99)	71.51 (30.43)	

**Table 2:** Adverse kidney outcomes. AKI = acute kidney disease; Stage 1 = increase in serum creatinine by ≥0.3 mg/dL within 48 h, or increase in serum creatinine 1.5 to 1.9 times baseline that is known or presumed to have occurred within the prior 7 days; Stage 2 = increase in serum creatinine to 2.0 to 2.9 times baseline; Stage 3 = increase in serum creatinine to 3.0 × baseline, or increase in serum creatinine to ≥4.0 mg/dL or initiation of kidney replacement therapy; KRT = Kidney replacement therapy: chronic dialysis (no patients with kidney transplantation).

Outcome Event	Event (*n*)	Denosumab (*n* = 4722)	Raloxifene (*n* = 4722)	*p* Value
		*n* (%)	*n*(%)	
AKI	1143	497 (10.53)	646 (13.68)	<0.0001
Stage 1	471	211 (4.47)	260 (5.51)	
Stage 2	114	46 (0.97)	68 (1.44)	
Stage 3 (including dialysis)	558	240 (5.08)	318 (6.73)	
KRT	61	23 (0.49)	38 (0.80)	0.0540
Chronic Dialysis	61	23 (0.49)	38 (0.80)	0.0540
eGFR reduction ≥30% of baseline	2986	1438 (30.45)	1548 (32.78)	0.0149

**Table 3:** Factors associated with mean change in eGFR over time.

	Model 1				Model 2			
Variables	β	95% CI		*p* Value	β	95% CI		*p* Value
Treatment								
Denosumab	1.78	(0.56, 3.01)		0.0042	1.68	(0.82, 2.54)		0.0001
Raloxifene	1.00	Reference			1.00	Reference		
Time	−0.05	(−0.07, −0.03)		<0.0001	−0.05	(−0.07,−0.03)		<0.0001
Treatment * time								
Denosumab * time	−0.33	(−0.36, −0.29)		<0.0001	−0.33	(−0.36, −0.29)		<0.0001
Raloxifene * time	1.00	Reference			1.00	Reference		
PDC (%) of treatment								
<60%					1.00	Reference		
≥60%					−0.66	(−1.51, −0.19)		0.1265
Age, year								
<65					1.00	Reference		
≧65					−11.14	(−12.04, −10.24)		<0.0001
Baseline eGFR								
≧60					1.00	Reference		
30–59.9					−34.14	(−35.04, −33.24)		<0.0001
<30					−59.84	(−61.37, −58.31)		<0.0001
AKI during follow-up time (yes vs. no)					−9.00	(−10.26, −7.73)		<0.0001

* Time (follow-up time) was the number of 3-month interval over the follow-up time.

## Data Availability

Data supporting reported results can be obtained from the corresponding author.

## Supplementary Materials

The following supporting information can be downloaded at: https://www.mdpi.com/article/10.3390/biomedicines10071494/s1, Figure S1: Estimated glomerular filtration rate (eGFR) trajectory of denosumab and raloxifene users during the study follow-up; Table S1: Codes for inclusion/exclusion criteria, baseline comorbid conditions, and medication use; Table S2: Comparative risk of adverse kidney outcomes between denosumab and raloxifene.
