# Supplementary material for: Comparative Adverse Kidney Outcomes in Women Receiving Raloxifene and Denosumab in a Real-World Setting
Source: Biomedicines. 2022 Jun 24;10(7):1494. doi: 10.3390/biomedicines10071494 (PMC9313089; doi:10.3390/biomedicines10071494)
Supplement: Supplementary file 1 [file biomedicines-10-01494-s001.zip › supplementary files/Supplement table 220406 (F).pdf]

Supplemental Table S1. Codes for inclusion/exclusion criteria, baseline comorbid conditions and medication use.

Inclusion/exclusion criteria and baseline comorbidity	
*Acute myocardial infarction	ICD-9-CM: 410<br>ICD-10-CM: I21, I22, I25
*Congestive heart failure	ICD-9-CM: 428<br>ICD-10-CM: I50
*Peripheral vascular diseases	ICD-9-CM: 441, 443.9, 785.4, V434<br>ICD-10-CM: I71, I73, I79
*Cerebral vascular accident	ICD-9-CM: 430, 431, 432, 433, 434, 435, 436, 437, 438<br>ICD-10-CM: I60, I61, I62, I63, I65, I66, I67, I69, G45, G46
*Dementia	ICD-9-CM: 290<br>ICD-10-CM: F01, F02, F05
*Pulmonary disease	ICD-9-CM: 490, 491, 492, 493, 494, 495, 496, 500, 501, 502, 503, 504, 505<br>ICD-10-CM: J40, J41, J42, J44, J43, J44, J45, J47, J60, J61, J62, J63, J64, J65, J66, J67
*Connective tissue disorder	ICD-9-CM: 517.1, 710, 714, 725<br>ICD-10-CM: M05, M06, M32, M33, M34, M35
*Peptic ulcer	ICD-9-CM: 531, 532, 533, 534<br>ICD-10-CM: K25, K26, K27, K28
*Liver diseases	ICD-9-CM: 571<br>ICD-10-CM: K70, K71, K73, K74
*Diabetes	ICD-9-CM: 250.0, 250.1, 250.2<br>ICD-10-CM: E10.1, E10.9, E11.0, E11.9, E13.1, E13.9
*Diabetes complications	ICD-9-CM: 250.4, 250.5, 250.6, 250.7, 250.8, 250.9<br>ICD-10-CM: E10.2, E10.3, E10.4, E10.5, E10.6, E10.8, E11.2, E11.3, E11.4, E11.5, E11.6, E11.8, E13.2, E13.3, E13.4, E13.5, E13.6, E13.8
*Paraplegia	ICD-9-CM: 342, 344.1<br>ICD-10-CM: G04.1, G81, G82
*Renal disease	ICD-9-CM: 582, 583, 585, 586, 588<br>ICD-10-CM: N01, N03, N05, N07, N18, N19, N25
*Severe liver diseases	ICD-9-CM: 572.2, 572.3, 572.4, 572.8<br>ICD-10-CM: K72.1, K72.9, K76.6, K76.7
Hypertension	ICD-9-CM: 401, 402, 403, 404, 405<br>ICD-10-CM: I10, I11, I12, I13, I15, N26.2
Hyperlipidemia	ICD-9-CM: 272<br>ICD-10-CM: E75.2, E75.3, E75.5, E75.6, E77, E78, E88.1, E88.2, E88.89
Thyroid function abnormal	ICD-9-CM: 242, 243, 244<br>ICD-10-CM: E00, E01, E03, E05, E89.0
Obstructive sleep apnea	ICD-9-CM: 780.51, 780.53, 780.57<br>ICD-10-CM: G47.30, G47.33, G47.39
Cancer	ICD-9-CM: 140-208<br>ICD-10-CM: Cxxx, Z51

Kidney transplantation	ICD-9-CM: V42.0<br>ICD-10-CM: Z94<br>**Billing code: 76020A, 76020B
End stage Kidney disease	. Hemodialysis:<br>ICD-9-CM/PCS: 39.95<br>ICD-10-PCS: 5A1D60Z, 5A1D00Z<br>**Billing code: 58001C, 58002CB, 58014C, 58018C, 58019C, 58020C, 58021C, 58022C, 58023C, 58024C, 58025C, 58027C, 58029C, 58030B<br>. Peritoneal dialysis:<br>ICD-9-CM/PCS: 54.98<br>ICD-10-PCS: 3E1M39Z<br>**Billing code: 58002C, 58009B, 58010A, 58010B, 58011A, 58011AB, 58017B
**Baseline medications**	**Anatomical Therapeutic Chemical (ATC) code**
Denosumab	M05BX04
Raloxifene	G03XC01
Oral anticoagulants	B01AA, B01AE, B01AF
Anti-platelet	B01AC
Aspirin	B01AC06
Statins	C10AA, C10BA, C10BX
Fibrates	C10AB, C10BA
Other lipid-lowering agents	C10AC, C10AX
Anti-diabetics	A10
ACEI/ARB/Aliskiren	C09AA, C09BB, C09CA, C09DA, C09DB, C09DX, C09XA
Diuretics	C03AA, C03DA, C03CB01, C03CA02
Bisphosphonates	M05BA02, M05BA03, M05BA06, M05BA07, M05BA08
Alendronate	M05BA04, M05BB03
Teriparatide	H05AA02
Calcitonin preparations	H05BA01, H05BA02, H05BA03
Calcium	A12AA04, A12AA12, A12AA91, A12AX
Vitamin D	A12AX, A11CC07
NSAID	M01AA, M01AX, M01AB, M01AC, M01AE, M01AG, M01AH

* Reference: Sundararajan V, Henderson T, Perry C, Muggivan A, Quan H, Ghali WA.(2004) New ICD-10 version of the Charlson comorbidity index predicted in-hospital mortality. J Clin Epidemiol;57(12):1288-94. doi:10.1016/j.jclinepi.2004.03.012.

Supplemental Table S2. Comparative risk of adverse kidney outcomes between denosumab and raloxifene.

	AKI			advanced AKI (stage 2/ 3)			eGFR reduction≥30%		
	**aHR**	**95% CI**	***p-value***	**aHR**	**95% CI**	***p-value***	**aHR**	**95% CI**	***p-value***
**Denosumab (vs raloxifene)**	**1.11**	**(0.97 –1.26)**	**0.1228**	**1.05**	**(0.88 –1.24)**	**0.6028**	**1.26**	**(1.16 –1.36)**	**<.0001**
**PDC (%)**									
<60%	1.00	(reference)		1.00	(reference)		1.00	(reference)	
≥60%	0.87	(0.76 –0.99)	0.0396	0.88	(0.74 –1.05)	0.1555	0.96	(0.89 –1.05)	0.3936
**Age group, years**									
<65	1.00	(reference)		1.00	(reference)		1.00	(reference)	
≥65	1.45	(1.18 –1.78)	0.0005	1.16	(0.89 –1.52)	0.2772	1.50	(1.35 –1.67)	<.0001
**Baseline eGFR, ml/min/1.73m^2**									
≧60	1.00	(reference)		1.00	(reference)		1.00	(reference)	
30-59.9	2.42	(2.07 –2.84)	<.0001	3.13	(2.42 –4.04)	<.0001	1.12	(1.03 –1.22)	0.0080
<30	10.58	(8.83 –12.67)	<.0001	26.05	(20.16 –33.66)	<.0001	1.43	(1.24 –1.65)	<.0001
**AKI (from index date to first event of eGFR reduction ≥30% or EOF)**							2.51	(2.24 –2.82)	<.0001
**Baseline comorbidities**									
Peripheral vascular diseases	1.19	(0.85 –1.68)	0.3141	0.96	(0.60 –1.55)	0.8727	0.98	(0.76 –1.26)	0.8460
Dementia	1.25	(0.99 –1.57)	0.0560	1.00	(0.73 –1.39)	0.9846	1.15	(0.98 –1.34)	0.0795
Pulmonary disease	1.17	(0.99 –1.38)	0.0649	0.97	(0.77 –1.23)	0.8079	1.14	(1.02 –1.26)	0.0161
Connective tissue disorder	1.22	(0.91 –1.63)	0.1856	1.16	(0.78 –1.72)	0.4597	1.12	(0.94 –1.34)	0.2115
Peptic ulcer	0.97	(0.85 –1.12)	0.6775	0.88	(0.73 –1.06)	0.1842	0.96	(0.88 –1.05)	0.4139
Liver diseases	1.09	(0.91 –1.31)	0.3468	0.99	(0.77 –1.27)	0.9452	1.10	(0.99 –1.23)	0.0914
Diabetes	1.33	(1.11 –1.60)	0.0018	1.28	(1.02 –1.61)	0.0315	1.23	(1.09 –1.38)	0.0005
Diabetes complications	1.49	(1.24 –1.78)	<.0001	1.72	(1.38 –2.16)	<.0001	1.17	(1.03 –1.33)	0.0158
Paraplegia	1.48	(0.95 –2.31)	0.0822	1.73	(0.91 –3.29)	0.0977	1.63	(1.25 –2.12)	0.0003
Renal disease	2.05	(1.76 –2.39)	<.0001	2.53	(2.10 –3.06)	<.0001	1.12	(0.99 –1.27)	0.0836
Severe liver diseases	2.21	(1.37 –3.56)	0.0011	1.38	(0.62 –3.04)	0.4277	2.15	(1.47 –3.16)	<.0001
Hypertension	1.20	(1.03 –1.40)	0.0188	1.24	(1.01 –1.52)	0.0429	1.20	(1.09 –1.31)	0.0001
Hyperlipidemia	0.79	(0.68 –0.93)	0.0033	0.81	(0.66 –0.99)	0.0412	0.92	(0.83 –1.01)	0.0847
Thyroid function abnormal	0.88	(0.55 –1.39)	0.5737	0.89	(0.47 –1.67)	0.7082	1.39	(1.11 –1.75)	0.0039
Obstructive sleep apnea	0.73	(0.40 –1.33)	0.3049	0.64	(0.28 –1.45)	0.2845	1.11	(0.79 –1.58)	0.5433
**Prior medications**									
Oral anticoagulants	1.41	(1.03 –1.91)	0.0300	0.94	(0.57 –1.54)	0.7923	1.26	(1.01 –1.55)	0.0366
Anti-platelet	1.04	(0.83 –1.30)	0.7248	0.96	(0.72 –1.27)	0.7503	1.06	(0.90 –1.23)	0.4943
Aspirin	0.98	(0.77 –1.24)	0.8498	0.96	(0.71 –1.30)	0.7773	1.05	(0.89 –1.23)	0.5886
Statins	0.92	(0.77 –1.09)	0.3186	1.04	(0.84 –1.29)	0.7262	0.94	(0.84 –1.05)	0.2585
Fibrates	1.05	(0.77 –1.41)	0.7760	1.11	(0.76 –1.62)	0.6052	0.93	(0.74 –1.16)	0.5039
Other lipid-lowering agents	0.85	(0.31 –2.30)	0.7475	0.30	(0.04 –2.17)	0.2323	0.83	(0.47 –1.46)	0.5146
Anti-diabetics	1.01	(0.82 –1.24)	0.9484	0.84	(0.65 –1.09)	0.1948	1.28	(1.11 –1.46)	0.0004
ACEI/ARB/Aliskiren	0.94	(0.80 –1.10)	0.4078	0.93	(0.76 –1.15)	0.5139	1.06	(0.95 –1.17)	0.3100
Diuretics	1.15	(0.94 –1.40)	0.1635	1.05	(0.80 –1.37)	0.7471	1.01	(0.88 –1.16)	0.9226
Alendronate	0.94	(0.80 –1.12)	0.5068	0.78	(0.61 –1.00)	0.0458	1.11	(1.01 –1.23)	0.0376
Teriparatide	1.42	(1.03 –1.95)	0.0335	1.32	(0.84 –2.08)	0.2261	1.31	(1.07 –1.60)	0.0084
Calcitonin preparations	0.79	(0.49 –1.29)	0.3539	0.67	(0.34 –1.32)	0.2498	0.91	(0.68 –1.23)	0.5550
Calcium	0.98	(0.83 –1.15)	0.8023	1.00	(0.81 –1.22)	0.9681	0.96	(0.86 –1.07)	0.4566
Vitamin D	0.93	(0.77 –1.12)	0.4133	0.91	(0.71 –1.15)	0.4249	1.02	(0.90 –1.15)	0.8132
NSAID	0.91	(0.80 –1.04)	0.1577	0.84	(0.71 –0.99)	0.0372	1.10	(1.01 –1.20)	0.0285

AKI: Acute kidney injury; EOF: End of follow up date; PDC: Proportion of days covered; ACEI/ARB: angiotensin-converting enzyme inhibitors/Angiotensin II receptor blockers; NSAID: Nonsteroidal anti-inflammatory drugs.
