# Supplementary figures and images for: Comparative Adverse Kidney Outcomes in Women Receiving Raloxifene and Denosumab in a Real-World Setting
Source: Biomedicines. 2022 Jun 24;10(7):1494. doi: 10.3390/biomedicines10071494 (PMC9313089; doi:10.3390/biomedicines10071494)

Trend of eGFR value

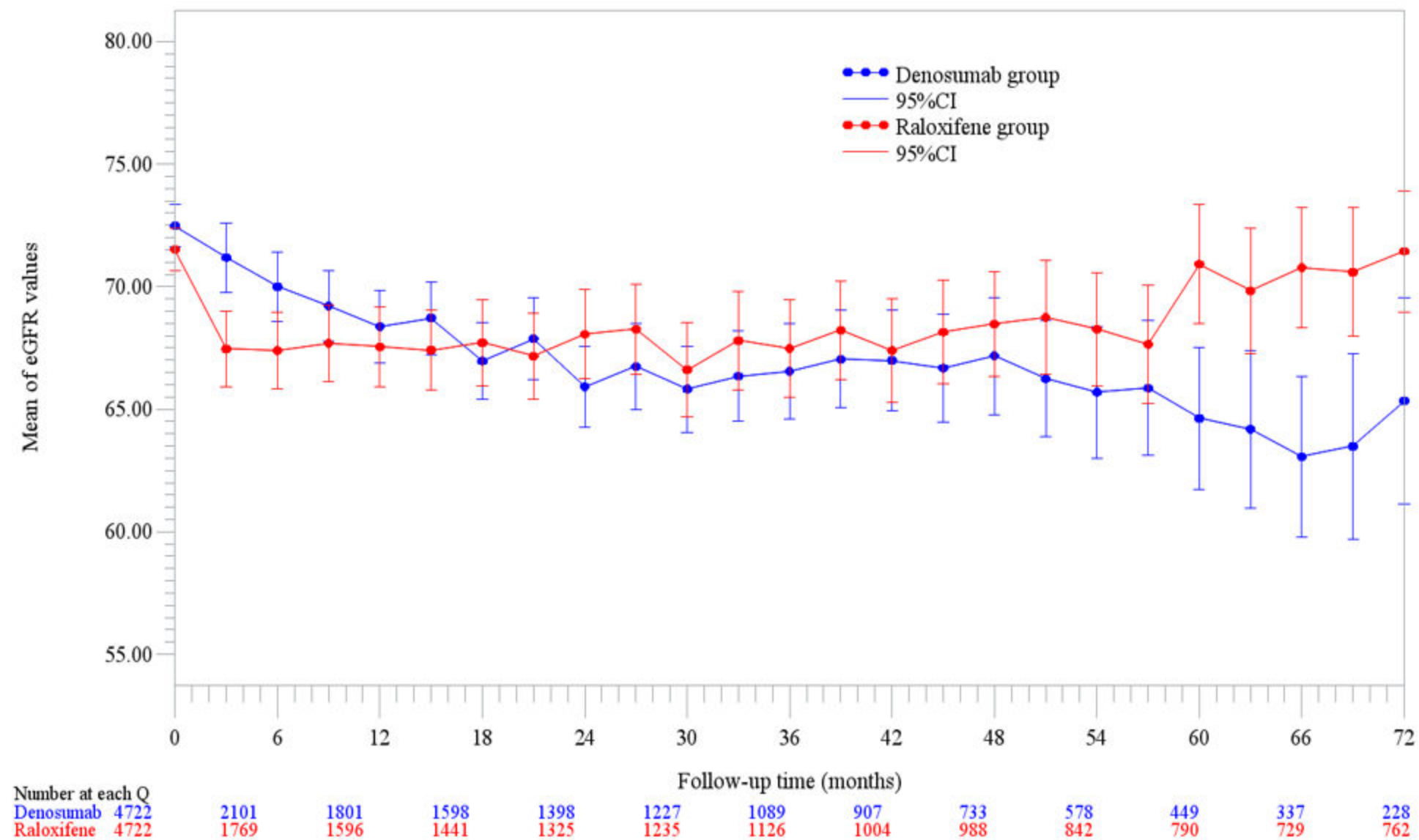

Supplement: Supplementary file 1 [file biomedicines-10-01494-s001.zip › supplementary files/Figure S1.pdf]
